# Hybrid structured CoNi_2_S_4_/Ni_3_S_2_ nanowires with multifunctional performance for hybrid capacitor electrodes and overall water splitting

**DOI:** 10.1039/d0ra05544a

**Published:** 2020-09-10

**Authors:** Xiaoyun Liu, Qian Li, Xin Zhang, Yueqiu Jiang

**Affiliations:** School of Science, Shenyang Ligong University Shenyang 110159 P. R. China liuxy@imr.ac.cn; Department of Development and Planning, Shenyang Ligong University Shenyang 110159 P. R. China yueqiujiang@sylu.edu.cn; School of Automobile and Transportation, Shenyang Ligong University Shenyang 110159 P. R. China

## Abstract

Rational design of electrode materials plays a significant role in potential applications such as energy storage and conversion. In this work, CoNi_2_S_4_/Ni_3_S_2_ nanowires grown on Ni foam were synthesized through a facile hydrothermal approach, revealing a large capacitance of 997.2 F g^−1^ and cycling stability with 80.3% capacitance retention after 5000 cycles. The device was prepared using CoNi_2_S_4_/Ni_3_S_2_//AC as the positive electrode and active carbon as the negative electrode, and delivered an energy density of 0.4 mW h cm^−3^ at a power density of 3.99 mW cm^−3^ and an excellent cycle life with 79.2% capacitance retention after 10 000 cycles. In addition, the hybrid CoNi_2_S_4_/Ni_3_S_2_ nanowires demonstrate excellent OER performance with low overpotential of 360 mV at 30 mA cm^−2^ and overpotential of 173.8 mV at −10 mA cm^−2^ for the HER, a cell voltage of 1.43 V, and excellent cycle stability.

## Abstract 2

Rational design of electrode materials plays a significant role in potential applications such as energy storage and conversion.

## Graphical abstract

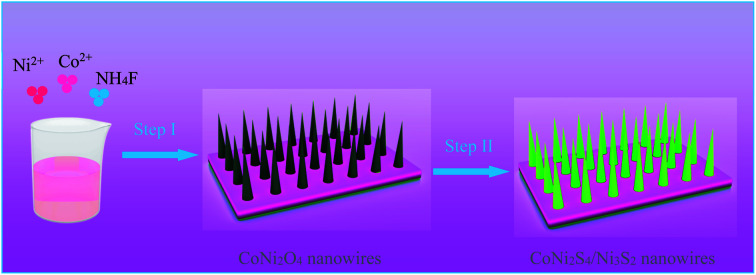

## 1. Introduction

In recent years, various transition metal-based catalysts such as phosphides, oxides, nitrides, carbides, and sulfides have been widely investigated as excellent electrochemical catalysts but the improvement of bi-functional electrocatalysts with excellent HER and OER activity is still difficult and slow to implement.^1–4^ To date, as demonstrated by experiments, electrocatalysts with vertically well-aligned arrays on 1D nanostructures consistently lead to improved HER and OER activity and promise efficient overall water splitting.^5,6^ Nanostructures possess distinctive advantages, including easier electrolyte diffusion and ionic transportation, better electrical connection with substrates, and offer larger surface area to provide contact between the electrode and electrolyte.^7^ Special nanostructures possess interconnected networks; these hierarchical heterostructures allow for efficient electron/ion transportation and accommodate volume variation nicely. Meanwhile, heterostructures consist of highly conductive materials as the “substratum” and transition metal oxides as the “superstratum”; thus, the combination of the two types of active materials create heterostructures that inherit advantages from both the “substratum” and “superstratum”.^8–10^ As highlighted in recent reports, Ni(OH)_2_/Ni_3_S_2_ nanostructure arrays on Ni foam show excellent electrochemical activity with a low overpotential of 270 mV (OER) at 20 mA cm^−2^ in 1.0 M KOH but exhibited a low HER activity (211 mV at 10 mA cm^−2^).^11^ Zhao *et al.* prepared nitrogen-doped carbon nanomaterials as efficient oxygen evolution electrocatalysts, which exhibited an overpotential of 380 mV at a current density of 10 mA cm^−2^ in alkaline media.^12^ An effective design of catalysts integrating HER and OER activity materials is the key for overall water splitting.^13^ Recently, benefits from cobalt include better redox activity under mild reaction conditions, modest overpotential, and the extremely high theoretical rich polymorphism of molybdenum, Co-based electrode materials have demonstrated vastly improved electrochemical performance.^14,15^

Among various electrode materials, spinel-structured CoNi_2_O_4_ has been investigated due to the high theoretical capacitance, high electrochemical performance and excellent electrocatalytic performance.^16,17^ For example, Tong *et al.* reported CoNi_2_O_4_ nanoneedles through a simple hydrothermal method. The as-prepared samples exhibited a specific capacitance of 1192 mF cm^−2^ at 2 mA cm^−2^ and cycle stability.^18^ Liu and co-workers prepared NiCo_2_O_4_ nanowires through a simple hydrothermal process; the as-prepared samples showed excellent electrocatalytic performance with an overpotential of 320 mV at 10 mA cm^−2^.^19^ However, single metal oxide electrode materials often present poor electrical conductivity, which limits the electrochemical performance. To improve the electrical conductivity, constructing a hybrid structure with metal sulfides has been considered an efficient method due to the improved electrical conductivity (about 100 times) over corresponding single metal oxides and binary materials.^20^ In the literature, Li *et al.* fabricated NiCo_2_S_4_@Co(OH)_2_ core–shell nanotube arrays, which showed an area capacitance of 9.6 F cm^−2^ at 2 mA cm^−2^.^21^ Jiang *et al.* prepared NiCo_2_S_4_@NiFe LDH heterostructures through a simple method, and the as-prepared electrocatalysts showed an overpotential of 201 mV and excellent cycle stability.^22^

Herein, we successfully synthesize CoNi_2_O_4_/Ni_3_S_2_ nanowires on Ni foam through a facile hydrothermal route. The as-fabricated samples deliver a high specific capacitance of 997.2 F g^−1^ at 1 A g^−1^ and cycle stability. In addition, the device exhibits an energy density of 0.4 mW h cm^−3^ at a power density of 3.99 mW cm^−3^ and an excellent cycle life with 79.2% capacitance retention after 10 000 cycles. As electrocatalysts, the hybrid CoNi_2_S_4_/Ni_3_S_2_ nanowires show excellent OER performance with a low overpotential of 360 mV and a small Tafel slope of 69.7 mV dec^−1^, and overpotential of 173.8 mV for the HER and Tafel slope of 98.8 mV dec^−1^ and excellent cycle stability.

## 2. Experimental section

### 2.1. Material preparation

Firstly, Ni foam was pre-treated *via* immersion in 0.1 M HCl, absolute ethanol and deionized water, respectively. Then, 1 mM Ni(NO_3_)_2_·6H_2_O, 2 mM Co(NO_3_)_2_·6H_2_O, 4 mM NH_4_F and 10 mM urea were dissolved in 60 mL deionized water. Then, the solution was transferred into an 80 mL Teflon-lined autoclave and kept at 120 °C for 8 h. Then, the samples were washed several times. Finally, the CoNi_2_O_4_ nanowires were obtained after calcination at 350 °C for 2 h (the average mass loading was 1.8 mg cm^−2^). Secondly, 0.35 g Na_2_S was dissolved into 40 mL deionized water and kept at 120 °C for 4 h. After cooling down to room temperature, the as-fabricated products were washed and dried at 60 °C. The average mass loading was 3.2 mg cm^−2^.

### 2.2. Materials characterization

The crystallographic structure of the as-fabricated samples was measured through X-ray diffraction. The morphology and structure of the samples were characterized by using scanning electron microscopy (SEM, Hitachi-4800) and high resolution transmission electron microscopy (HRTEM, JEM-2100 PLUS). X-ray photoelectron spectroscopy (XPS) measurements were conducted to investigate the element composition using an ESCALAB250 with Al Kα sources.

### 2.3. Electrochemical measurement

Electrochemical measurements of the as-prepared products including cyclic voltammetry (CV) curves, galvanostatic charge–discharge (GCD) and electrochemical impedance spectroscopy (EIS) were carried out by a CHI660e electrochemical workstation in a three-electrode system in 3.0 M KOH solutions. The as-fabricated samples (*d* = 1 cm) were used as the working electrode, Pt foil served as the counter electrode and Hg/HgO as the reference electrode. Electrochemical impedance spectroscopy (EIS) measurements were conducted in a frequency range from 0.01 to 100 kHz at a 5 mV open circuit potential. The specific capacitance of the as-prepared samples was calculated from GCD curves *via* the following equations;

1 *C*_s_ = *I*Δ*t*/*mV*

where *C*_s_, *I*, Δ*t*, *V* and *m* are the specific capacitance (F g^−1^), current density (A), discharge time (s), voltage (*V*) and mass loading (mg), respectively.

### 2.4. Fabrication of the hybrid battery

A hybrid battery was assembled with active carbon as the negative electrode, the as-fabricated CoNi_2_S_4_/Ni_3_S_2_ nanowires as the positive electrode and PVA–KOH gel as the electrolyte. PVA–KOH gel as the electrolyte was prepared as follows: 3 g PVA was added in 25 mL deionized water at 65 °C. Then, 3 g KOH was dissolved in 5 mL deionized water and added to the above solution.

### 2.5. Electrocatalytic performance of the hybrid battery

OER and HER performance tests of the as-prepared device were conducted in the three-electrode system. The as-fabricated samples were the working electrodes, and Ag/AgCl was the reference electrode. A graphite rod was the counter electrode. Overall water splitting was measured in a two-electrode system. All potentials were converted to reversible hydrogen electrode (RHE) though the Nernst equation *E*_RHE_ = *E*_Ag/AgCl_ + 0.197 + 0.059 × pH, where *E*_Ag/AgCl_ is the measured potential.

## 3. Results and discussion

**Fig. 1:**
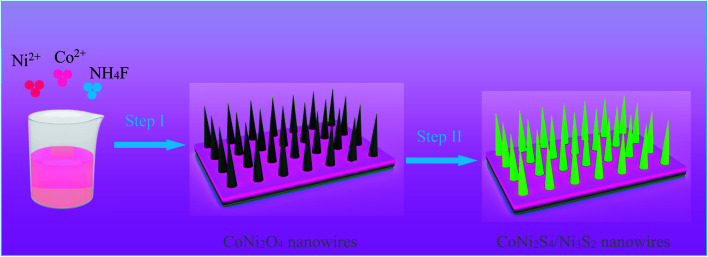
Schematic illustration of the fabrication process of CoNi_2_S_4_/Ni_3_S_2_ nanowires.

Fig. 1 shows the synthesis diagram of the hybrid CoNi_2_S_4_//Ni_3_S_2_ nanowires through a two-step hydrothermal process. Firstly, CoNi_2_O_4_ products were fabricated on the surface of Ni foam through a simple hydrothermal process. Then, the hybrid CoNi_2_S_4_/Ni_3_S_2_ products were prepared by an *in situ* vulcanization process.

**Fig. 2:**
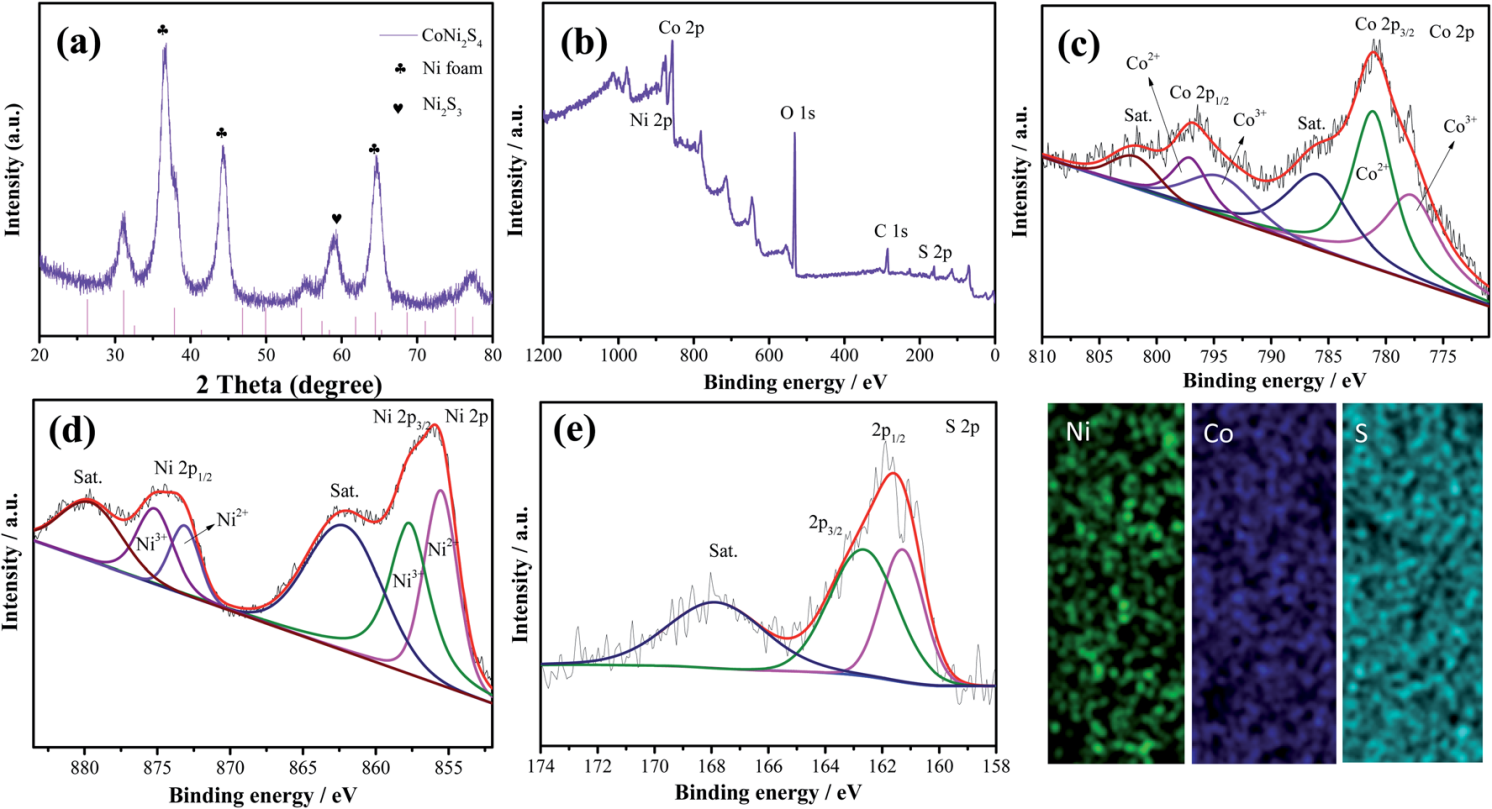
(a) XRD pattern of the as-fabricated CoNi_2_S_4_/Ni_3_S_2_ nanowires. (b) Survey spectra of the samples (c) Co 2p, (d) Ni 2p and (e) S 2p. (f) Elemental mapping.

Fig. 2a shows the crystal structure of the samples; the diffraction peaks at 2*θ* values of 44.4, 51.6 and 76.1 degrees can be indexed to Ni foam (JCPDS no. 04-0850). The diffraction peaks at 2*θ* values of 31.4, 38.2, 50.3, 55.0 and 64.8 degrees could be ascribed to the (311), (400), (511), (440) and (533) crystal planes of spinel CoNi_2_S_4_ phase (JCPDS no. 24-0334). In addition, the diffraction peaks at 31.1, 37.8, 44.3, 50.1 and 55.2 degrees could be indexed to the (110), (003), (202), (211) and (300) crystal planes of Ni_3_S_2_ (JCPDS no. 44-1418), respectively, which can be attributed to the vulcanization of the Ni foam. XPS analyses were used to further investigate the chemical composition and valence. Fig. 2b shows the XPS full survey spectrum of the CoNi_2_S_4_//Ni_3_S_2_ nanowires, and the products consisted of Ni, Co and S. The Co 2p spectrum (Fig. 2c) contains two spin–orbit doublets and two shakeup satellites, the binding energy at 779.2 and 795.1 eV could be ascribed to Co^3+^, and the binding energies at 780.9 and 795.3 eV belong to the Co^2+^ phase.^23^ In addition, the satellite peaks reveal that Co^3+^ is present.^24^Fig. 2d shows the Ni 2p spectrum, which can be divided into two spin–orbit doublets and two shakeup satellites. The binding energies at 854.8 and 871.6 eV can be assigned to Ni^3+^ and 854.3 and 871.2 eV are Ni^2+^.^25^ The sharp intense satellite peaks indicate that Ni is present as Ni^2+^.^26^ The S 2p spectrum is presented in Fig. 2e. The peak at 161.2 eV is S 2p_1/2_ and the peak at 162.3 eV belongs to a typical metal–sulfur bond, which improves the electrochemical activity of the as-prepared samples.^27,28^ Energy-dispersive X-ray spectrometry (EDX) measurements of the as-prepared CoNi_2_S_4_/Ni_3_S_2_ nanowires indicate that the as-fabricated products are composed of Ni, Co and S, as shown in Fig. 2f, and the three elements are uniformly distributed on the surface of the as-prepared samples, revealing that CoNi_2_S_4_/Ni_3_S_2_ nanowires are uniformed deposited on the surface of Ni foam.

**Fig. 3:**
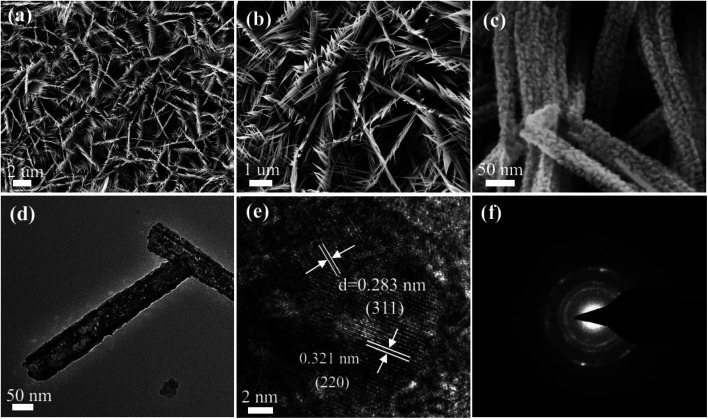
SEM images of the as-fabricated CoNi_2_S_4_/Ni_3_S_2_ nanowires. (a and b) Low magnification and (c) high magnification images. (d) TEM images. (e) HRTEM images. (f) SAED images.

The morphology characteristics of the as-synthesized CoNi_2_S_4_/Ni_3_S_2_ nanowires were first characterized through SEM. From the low magnification SEM images in Fig. 3a, it is clearly found that CoNi_2_S_4_/Ni_3_S_2_ nanowires are uniformly located on the surface of Ni foam. From Fig. 3b, it can be found that adjacent nanowires are connected to each other to form a network structure. Fig. 3c presents high magnification SEM images, indicating that the as-prepared CoNi_2_S_4_/Ni_3_S_2_ nanowires possess an average diameter of 75 nm. TEM measurements were used to further characterize the microstructures of CoNi_2_S_4_/Ni_3_S_2_ nanowires. Fig. 3d exhibits the low-magnification TEM images of the as-fabricated samples, in which a single nanowire is made of some tiny nanoparticles and the samples show the average diameter of 70 nm. The HRTEM image shows that the lattice spacing of 0.321 nm matches well with the (220) plane of Ni_3_S_2_ phase, and the lattice spacing of 0.283 nm can be indexed to the (311) plane of CoNi_2_S_4_ spinel structure. As can be seen from Fig. 3e, it can further confirmed that the as-prepared samples contain CoNi_2_S_4_ and Ni_3_S_2_ phases, which is consistent with the XRD patterns. The SAED pattern illustrated in Fig. 3f, shows that the as-fabricated samples present polycrystalline features that possess more active sites and energy barriers for adsorbing water molecules.

**Fig. 4:**
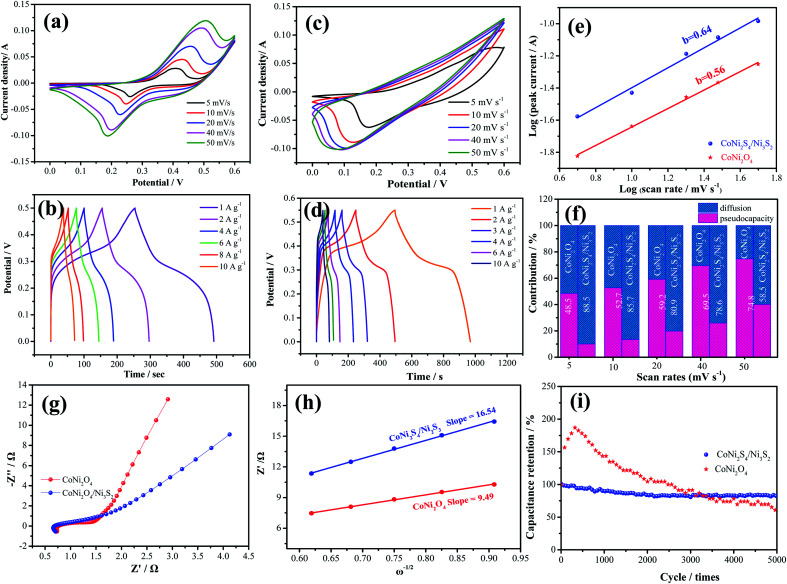
Electrochemical performance measurements. (a) CV curves of CoNi_2_O_4_, (b) GCD curves of CoNi_2_O_4_ samples, (c) CV curves of CoNi_2_S_4_/Ni_3_S_2_, (d) GCD curves of CoNi_2_S_4_/Ni_3_S_2_, (e) *b* values of two kinds of electrode materials, (f) capacitance contribution ratio, (g) Nyquist plots, (h) *Z*′ as a function of *ω*^−1/2^ plot in low frequency, (i) cycling stability tests.

To investigate the electrochemical performances of the as-prepared samples, Fig. 4a exhibits the CV curves of CoNi_2_O_4_ samples at scan rates from 5 to 50 mV s^−1^. It is seen that CV curves show a pair of redox peaks, demonstrating that the products possess typical pseudocapacitive characteristics. Fig. 4b shows the GCD curves of the CoNi_2_S_4_/Ni_3_S_2_ samples, and it is seen that the samples possess a specific capacitance of 997.2 F g^−1^ at a current density of 1 A g^−1^. CV curves of the hybrid CoNi_2_S_4_/Ni_3_S_2_ samples are depicted in Fig. 4c, and it is observed that the integrated areas increase as the scan sweep increases, indicating that the as-fabricated samples possess a fast ion and electron transfer rate. Based on previous reports, Ni foam contributes little to the capacitance.^29^Fig. 4d shows the GCD curves of the as-prepared electrode at different current densities from 1 A g^−1^ to 10 A g^−1^. It is found that the as-fabricated samples exhibit a high specific capacitance of 997.2 F g^−1^ at 1 A g^−1^. To further analyze the electrochemical kinetics of the as-prepared samples, the capacitance can be calculated according to following equation:

2 *i* = *av*^*b*^

where *a* and *b* are constants. The *b* value of 0.5 and 1.0 represent capacitive behavior and battery behavior, respectively. The *b* value (Fig. 4e) for reduction peaks of CoNi_2_O_4_ and CoNi_2_S_4_/Ni_3_S_2_ electrodes is 0.64 and 0.56, respectively. In addition, the capacitive contribution ratio can be obtained through the following equation:^30^

3 *i* = *k*_1_*ν* + *k*_2_*ν*^1/2^

where *i*, *ν*, *k*_1_ and *k*_2_ represent the current, scan rates and the constant, respectively. Fig. 4f shows the calculated pseudocapacitance and diffusion-controlled capacitance at scan rates from 5 to 50 mV s^−1^. It is seen that the contribution of diffusion-controlled capacity gradually decreases with increasing scan rate, indicating that the samples show fast transmission for OH^−^. At the same time, the pseudocapacitance arrives at the highest value of 74.8% at 50 mV s^−1^. EIS is used to further investigate the electrochemical performance, as shown in Fig. 4g. It is obvious that the intercept at the real axis shows the *R*_s_ value of CoNi_2_S_4_/Ni_3_S_2_ nanowires of 0.61 Ω. The underlying mechanism of differences in the capacitance performance of different samples was explored in the corresponding Nyquist curves, as shown in the inset of Fig. 4g. The CoNi_2_S_4_/Ni_3_S_2_ electrode showed lower contact resistance and charge transfer resistance than the CoNi_2_O_4_ nanowires, further confirming that the hybrid structure possesses excellent electrochemical performance. Simultaneously, in order to study the reaction kinetics in the low frequency region, the spectrum can be fit using the following equation:

4 *Z* = *R*_s_ + *R*_ct_ + *σ*_w_*ω*^−1/2^

where *σ*_w_ is the Warburg factor and *ω* is angular frequency. *Z* can be attributed to the diffusive resistance of OH^−^. The values of CoNi_2_O_4_ and CoNi_2_S_4_/Ni_3_S_2_ electrodes are 16.54 and 9.49, respectively, indicating that CoNi_2_S_4_/Ni_3_S_2_ samples possess a fast transmission for OH^−^ (Fig. 4g). The cycling stability of the as-fabricated samples is measured by GCD measurements, as illustrated in Fig. 4i, revealing that the as-prepared CoNi_2_S_4_/Ni_3_S_2_ sample shows more excellent stability with 80.3% capacitance retention than CoNi_2_O_4_ samples (64.2%) after 5000 cycles.

**Fig. 5:**
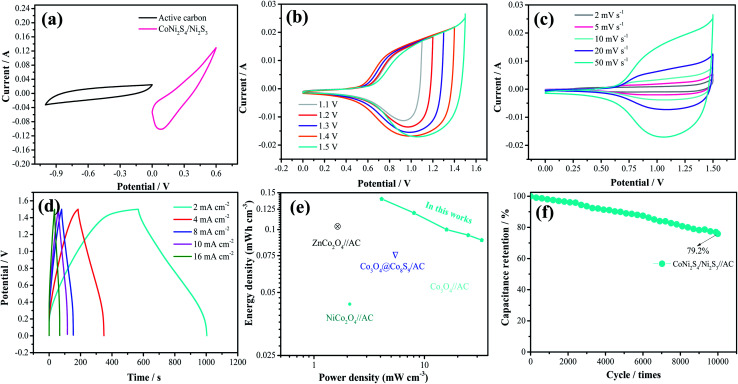
Electrochemical performance characteristics of the as-fabricated device. (a) The CV comparison of CoNi_2_S_4_/Ni_3_S_2_//AC, (b) CV curves at different potential window, (c) CV curves, (d) GCD curves, (e) Ragone plots, (f) cycling stability measurements.

To further investigate the practical application of the as-fabricated electrode, the device is assembled with the CoNi_2_S_4_/Ni_3_S_2_ nanowires electrode as the positive electrode and active carbon as the negative electrode. The composition of CV curves of the CoNi_2_S_4_/Ni_3_S_2_ nanowires and active carbon at a current density of 50 mV s^−1^ are depicted in Fig. 5a. It is found that the electrode materials exhibit single potential windows of −1.0–0 V and 0–0.6 V, respectively, indicating that the device can reach a total potential of 1.6 V. Fig. 5b exhibits the CV curves of the fabricated CoNi_2_S_4_/Ni_3_S_2_ nanowires//AC device at scan rates from 2 to 50 mV s^−1^. It can be seen that the device can work in a voltage window of 0–1.5 V. With increasing scan rates, the CV shapes are maintained, indicating that the capacitors possess excellent rate performance. CV curves of the CoNi_2_S_4_/Ni_3_S_2_ nanowires//AC device with different potential windows at a scan rate of 50 mV s^−1^ are collected to investigate the extended working voltage, as illustrated in Fig. 5c, and the device shows a stable capacitive behavior with electric double-layer capacitive properties even at a voltage of up to 1.5 V. Fig. 5d shows the GCD measurement of the device, which showed that the areal capacitance of the CoNi_2_S_4_/Ni_3_S_2_ nanowires//AC device reaches 136.05 mF cm^−2^ at a current density of 2 mA cm^−2^. As the current density increased to 10 mA cm^−2^, the areal capacitance was maintained at 56 mF cm^−2^. The Ragone plots of the asymmetric supercapacitors are shown in Fig. 5e. The CoNi_2_S_4_/Ni_3_S_2_ nanowires//AC supercapacitors deliver a maximum energy density of 0.4 mW h cm^−3^ at a power density of 3.99 mW cm^−3^, which is comparable to previously reported ASC devices.^31–34^ The long-term cycling stability of the CoNi_2_S_4_/Ni_3_S_2_ nanowires//AC device is also evaluated by repeating charge–discharge measurements, as shown in Fig. 5f. After 10 000 cycles, there is still 90% retention of the original capacitance at a current density of 5 mA cm^−2^.

**Fig. 6:**
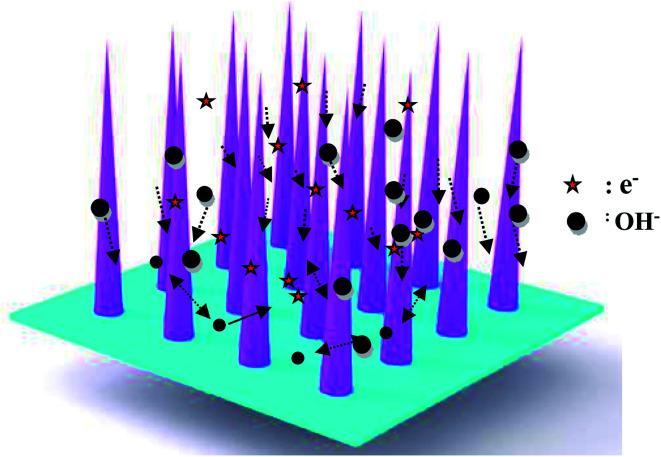
Electrochemical mechanism diagram of the CoNi_2_S_4_/Ni_3_S_2_ samples.

The as-fabricated samples possess excellent cycle stability, which can be ascribed to the following aspects, as shown in Fig. 6. First, the as-fabricated CoNi_2_S_4_/Ni_3_S_2_ nanowires are directly on Ni foam, which provides a lot of active sites. Secondly, the hybrid CoNi_2_S_4_/Ni_3_S_2_ structure alleviates the volume change during the electrochemical reaction process. Finally, the adjacent nanowires possess enough reaction space, which leads to a rapid electrochemical reaction.

**Fig. 7:**
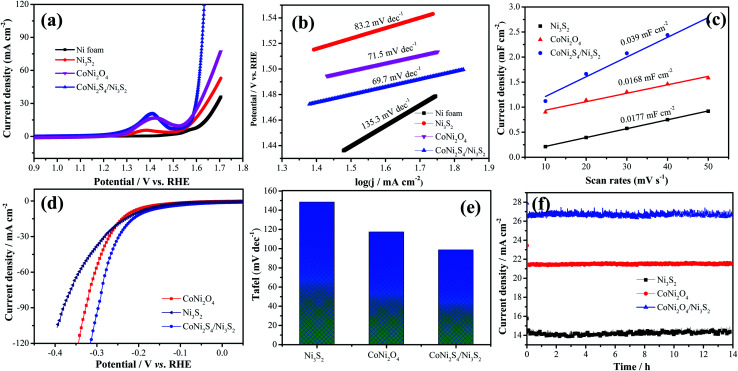
(a–c) OER of the as-prepared catalysts, (a) LSV curves, (b) Tafel, (c) electric double layer capacitor value, (d) LSV curves for HER, (e) Tafel for HER, (f) cycle stability.

The electrocatalytic performance of the as-prepared catalysts is estimated through LSV and Tafel curves in 1.0 M KOH solution. As shown in Fig. 7a, the hybrid CoNi_2_S_4_/Ni_3_S_2_ nanowires show a lower overpotential of 360 mV compared to that of CoNi_2_O_4_ nanowires (380 mV), Ni_3_S_2_ samples (410) and Ni foam (459 mV) at a current density of 30 mA cm^−2^, which demonstrates that CoNi_2_S_4_/Ni_3_S_2_ nanowires possess better electrocatalytic performance. To give insight into the OER kinetic mechanism, Fig. 7b presents the Tafel slope of the catalyst. Hybrid CoNi_2_S_4_/Ni_3_S_2_ nanowires exhibit a small Tafel slope of 69.7 mV dec^−1^, which is lower than that of the CoNi_2_O_4_ nanowires (71.5 mV dec^−1^), Ni_3_S_2_ samples (83.2 mV dec^−1^) and Ni foam (135.3 mV dec^−1^). The low Tafel slope increases the charge transfer rate and provides active sites for OH adsorption.^35^ To further study the outstanding OER activity, the electrochemical active surface area (ECSA) was measured through the double-layer capacitance (*C*_dl_). The value of the electrochemical double layer capacitance is shown in Fig. 7c. CoNi_2_S_4_/Ni_3_S_2_ nanowires exhibit a higher ECSA (0.039 mF cm^−2^) than CoNi_2_O_4_ nanowires (0.0168 mF cm^−2^) and Ni_3_S_2_ samples (0.0177 mF cm^−2^). The HER performance of the as-prepared samples is also tested at 5 mV s^−1^. The comparison of the LSV curves of catalysts is depicted in Fig. 7d. It is clearly found that hybrid CoNi_2_S_4_/Ni_3_S_2_ nanowires exhibit a small Tafel slope of 173.8 mV at −10 mA cm^−2^, which is lower than that of CoNi_2_O_4_ nanowires (192.6 mV) and Ni_3_S_2_ samples (210.8 mV). Fig. 7e shows the corresponding Tafel plots, which shows that hybrid CoNi_2_S_4_/Ni_3_S_2_ nanowires exhibit a small Tafel slope of 98.8 mV dec^−1^, which is lower than the CoNi_2_O_4_ nanowires (117.2 mV dec^−1^) and Ni_3_S_2_ samples (148.9 mV dec^−1^). The Tafel slope of CoNi_2_S_4_/Ni_3_S_2_ nanowires is smallest, revealing the fast reaction kinetics. Since the Tafel slope is in the range from 40 to 120 mV dec^−1^, this implies that the HER reaction of CoNi_2_S_4_/Ni_3_S_2_ nanowires obey the Volmer–Heyrovsky mechanism, and the Volmer step is rate-determining.^36^ The long-term stability measurements of the three electrode materials are conducted at a constant potential for 13 h, as shown in Fig. 7f. The results demonstrate that the as-fabricated catalysts present excellent cycle stability.

**Fig. 8:**
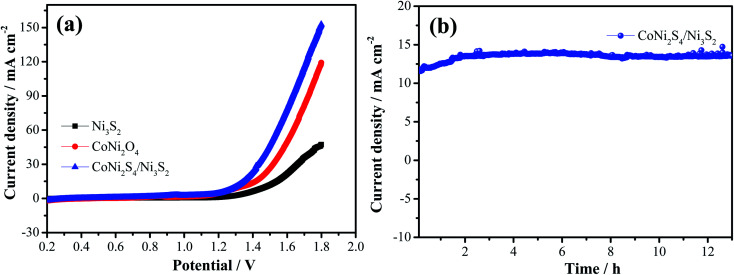
Overall water splitting of the as-prepared samples. (a) LSV curves, (b) cycle stability.

To further investigate the potential practical applications, overall water splitting tests are conducted through a two-electrode system with the hybrid structured catalysts as both the cathode and anode. Fig. 8a presents the LSV curve of overall water splitting with a scan rate of 5 mV s^−1^. It is seen that hybrid CoNi_2_S_4_/Ni_3_S_2_ samples show a small cell voltage of 1.43 V at 30 mA cm^−2^, which is lower than that of CoNi_2_S_4_ nanowires (1.51 V) and Ni_3_S_2_ samples (1.65 V). In order to evaluate the stability of the as-prepared catalysts, chronoamperometry measurements were conducted, as depicted in Fig. 8b. It was found that the hybrid structured CoNi_2_S_4_/Ni_3_S_2_ samples possess excellent long durability. At the same time, a large number of bubbles were formed from both the cathode and anode as the electrocatalytic test proceeded.

## 4. Conclusion

In summary, hybrid structured CoNi_2_S_4_/Ni_3_S_2_ nanowires were fabricated through a facile two-step hydrothermal method. The as-prepared products directly act as a supercapacitor electrode with a specific capacitance of 997.2 F g^−1^ at 1 A g^−1^ and cycle stability of 80.3% retention after 5000 cycles. The asymmetric supercapacitor shows a high energy density of 0.4 mW h cm^−3^ at a power density of 3.99 mW cm^−3^ and cycling stability of 79.2% retention after 10 000 cycles. The as-fabricated CoNi_2_S_4_/Ni_3_S_2_ nanowires also exhibit excellent electrocatalytic performance with a low overpotential of 360 mV for the OER and a small Tafel slope of 98.8 mV dec^−1^ and a cell voltage of 1.43 V.

## Conflicts of interest

The authors declare no conflict of interest.

## Supplementary Material
